# Association of calcaneal quantitative ultrasound parameters with metabolic syndrome in middle-aged and elderly Chinese: a large population-based cross-sectional study

**DOI:** 10.1186/1472-6823-14-14

**Published:** 2014-02-14

**Authors:** Min Sun, Mengdie Cao, Qi Fu, Zhenxin Zhu, Chuchen Meng, Jia Mao, Yun Shi, Yu Duan, Wei Tang, Xiaoping Huang, Wei He, Tao Yang

**Affiliations:** 1 Department of Endocrinology, First Affiliated Hospital of Nanjing Medical University, 300 Guangzhou Road, Nanjing, Jiangsu 210029, China

**Keywords:** Metabolic syndrome, Osteoporosis, Calcaneal quantitative ultrasound, Fragile fractures

## Abstract

**Background:**

The possible association between metabolic syndrome (MS) and bone mineral density (BMD) has been highlighted recently. However, the exact effects of MS on calcaneal quantitative ultrasound (QUS) parameters remains uncertain. The aim of this study was to assess the impact of MS states, different componets of MS, as well as the number of MS componets on QUS.

**Methods:**

A total of 7489 Chinese adults aged 40 years or older in Nanjing were enrolled in this cross-sectional study. MS was defined according to recommendations generated by the International Diabetes Federation (IDF) in 2005. QUS was measured for each participant.

**Results:**

The prevalence of MS was 34.6% in men and 42.8% in women (over 40 years old). In postmenopausal women with MS, 25-hydroxyvitamin D[25(OH)D], age adjusted quantitative ultrasound index (QUI) and broadband ultrasound attenuation (BUA) were all lower than those without (p < 0.001, p = 0.023, p = 0.021, respectively), the difference of QUI and BUA disappeared after adjustment for body mass index (BMI) and waist circumference (WC). In stepwise analysis, BMI, WC, high density lipoprotein cholesterol (HDL-C) and fasting plasma glucose (FPG) were related to QUS (p < 0.05). The number of MS components had no influence on QUS. Fragile fracture incidence was higher in women with MS (6.8% VS. 5.3%, P = 0.034).

**Conclusion:**

Chinese postmenopausal women with MS have worse BMD measured by QUS and more chances to develop osteoporotic fractures than the controls, which partially due to central obesity as well as vitamin D deficiency. People having less central obesity, higher FPG or HDL-C are less likely to have bone mineral loss.

## Background

Metabolic syndrome is characterized by a constellation of cardiovascular risk factors that includes high abdominal adiposity, blood pressure, glucose and triglycerides (TG) and low HDL-C levels [1]. It has become increasingly common worldwide, especially in old population, and it is considered to be highly associated with increased risk of developing cardiovascular disease and type 2 diabetes mellitus (T2DM) [2]. The prevalence of MS in asymptomatic US adults 40 years and older was 22.8% [3]. The prevalence of MS in Chinese older than 20 years was 12%-21% diagnosing by National Education Cholesterol Program Adult Treatment Panel-III (NECP-ATPIII) criterion [4].

Osteoporosis is another common metabolic bone disease characterized by prominent skeletal fragility (due to reduction in both bone quantity and quality), leading to an increased risk of developing spontaneous fractures and chronic pain [5]. The prevalence of osteoporosis in old age groups is very high. A nationwide epidemiological survey from 1997 to 1999 showed that the prevalence of osteoporosis in Chinese over 40 years old was 19.9% for women and 11.5% for men, BMD of vertebra and femur neck measured by dual energy X-ray absorptiometry (DXA) [6].

Recently, more evidence has accumulated for a possible link between MS and osteoporosis, but the results from different studies are inconsistent. Some studies suggested that MS might be a risk factor for osteoporosis [7,8], while others concluded that MS can protect against osteoporotic fractures [9,10]. Most of the previous studies about the relationship are based on the BMD derived by DXA. QUS, another method to evaluate BMD, can reflect both bone mass and bone quality [11]. According to a study in Germany, QUS and DXA are equally strongly associated with risk factors for osteoporosis [12]. On the other hand, although both DXA and QUS are good predictors for osteoporotic fractures, they still differ in several aspects. Compared to DXA, QUS is more convenient, easily available and lack of ionizing radiation, so it’s a simple and inexpensive method for screening osteoporosis, especially in population-based studies. Until now, only few surveys have addressed the association between MS and QUS and the results are controversial [13-16]. Whether MS has a strong impact on QUS, whether different components of MS contribute diversely to the QUS are worth further investigation.

In the present cross-sectional study, we had the following specific aims: 1) to compare the QUS parameters in individuals with and without MS in Chinese population; 2) to determine the association of the QUS parameters with the components of MS respectively, as well as with the number of MS components; 3) to study the relationship between the osteoporotic fracture and MS states as well as QUS measurements.

## Methods

### Study participants

People older than 40 years old who lived in Gulou, Nanjing, China were invited to participate in this community-based project. A total of 9982 people were recruited, from June to December 2011. The study was approved by the ethics committee of The First Affiliated Hospital with Nanjing Medical University. Totally 2493 individuals who met the following criteria were excluded: 1) individuals had not finish the questionnaire or examination; 2) those taking calcium, vitamin D or other medication for osteoporosis; 3) having received sexual hormone replacement therapy or other medications affecting QUS, such as glucocorticosteroid; 4) has a history of hematologic disease, rheumatoid arthritis or systemic lupus erythematosus, etc. Finally, 7489 individuals were involved in our study.

A standard questionnaire was used for collection of all the information about health states, medications, and lifestyle. The smoking or alcohol consumption habit was classified as never, current (smoking or consuming alcohol regularly in the past 6 months), or ever (cessation of smoking or alcohol consumption for more than 6 months). The type, amount, and frequency of alcohol consumption were also recorded.

Physical examinations were performed by professional physicians. The BMI was calculated as body weight (kg) divided by squared body height (m^2^). WC was measured in centimeters with a flexible tape all around the body at the level of navel. Blood pressure was measured with a sphygmomanometer and was taken as the mean of 3 consecutive measurements after at least 5 minutes of rest.

### Laboratory measurements

After a requested 12-hour fast, morning blood samples of every participants were obtained from an antecubital vein for measurements of FPG, serum HDL-C, low density lipoprotein cholesterol (LDL-C), TG and total cholesterol (TC). Plasma glucose was measured by hexokinase method. HDL-C, LDL-C, TG and TC were quantified using chemiluminescence assays on the auto-analyzer (Modular E170; Roche). Serum 25(OH)D was assessed by enzyme immunoassay (IDS, UK).

### MS definition

We used recommendations generated by IDF in 2005 for MS definition. Subjects were considered as MS if they had central obesity (WC ≥ 90 cm in Chinese men and ≥80 cm in Chinese women, or BMI over 30 kg/m^2^) plus at least two abnormalities from the following four components: TG > 1.7 mmol/L or receiving specific treatment for this lipid abnormality; HDL-C < 1.03 mmol/L in men and <1.29 mmol/L in women or receiving specific lipid-lowing therapy; SBP ≥ 130 mmHg or DBP ≥ 85 mmHg or having treatment of previously diagnosed hypertension; FPG ≥ 5.6 mmol/L or previous history of type 2 diabetes.

### Calcaneal QUS assessment

Calcaneal QUS was measured at the participant’s dominant foot by experienced operators with the Sahara Clinical Sonometer (Hologic, Bedford, MA, USA). BUA (dB/MHz) and speed of sound (SOS) (m/s) were recorded and the QUI was calculated from BUA and SOS. Daily calibration was performed before measurement according to the manufacturer’s recommendations.

### Identification of fragile fractures

According to the questionnaire, all fractures were self-reported and collected. Fractures were classified as either minor (e.g. fall from standing height or less) or sever trauma (e.g. resulting from a harder fall, a car accident or other severe trauma). Fragile fractures were defined as fractures that occur with a minor trauma after 25 years of age.

### Statistical analysis

Analysis was performed using SPSS 17.0 for Windows (Chicago, IL, USA). Student’s *t* test and Х^2^ test were used to compare the difference between two groups for continuous variables and categorical variables, respectively. To compare the QUS parameters adjusting for covariates including age, BMI, WC, daily exercise, current smoking and current alcohol intake, covariance analysis was performed. The relationship between QUS parameters with the components of MS was determined using stepwise multiple linear regressions. Spearman and partial correlation was also used to analysis the connection between number of MS components and QUS parameters. Logistic regression models were constructed to analyse the association between osteoporosis fractures and MS states, as well as QUS parameters.

## Results

### Baseline characteristics and laboratory parameters

Finally, a total of 7489 individuals were eligible for analysis in this study, of them there were 2814 males and 4675 females, including 983 premenopausal women and 3692 postmenopausal women. The prevalence of MS was 34.6% in men and 42.8% in women. Table 1 shows the baseline characteristics of the participants, grouped by sex, menstrual states and MS states. Participants with MS had higher weight, BMI, WC, TG and lower HDL-C compared with those without MS. However, regarding regular exercise, current smoking and current alcohol consumption, no difference was found between groups with or without MS.

**Table 1:** Baseline characteristics of the participants, grouped by sex, menstrual states and MS states

	**Males (n = 2814)**		**Females (n = 4675)**			
			**Premenopausal (n = 983)**		**Postmenopausal (n = 3692)**	
MS	Y (n = 973)	N (n = 1841)	Y (n = 237)	N (n = 746)	Y (n = 1763)	N (n = 1929)
Age (yrs)	59.4 ± 9.2	58.9 ± 9.1	47.7 ± 4.0***	46.0 ± 3.8	61.2 ± 7.5***	57.7 ± 7.6
Ht (cm)	169.1 ± 5.9***	167.4 ± 6.2	159.1 ± 5.3	158.5 ± 5.2	156.5 ± 5.6	156.6 ± 5.4
Wt (kg)	77.4 ± 8.2***	65.8 ± 8.4	67.3 ± 8.8***	57.6 ± 7.2	64.3 ± 8.4***	56.2 ± 7.5
BMI (kg/m2)	27.1 ± 2.4***	23.5 ± 2.5	26.6 ± 3.0***	22.9 ± 2.6	26.3 ± 3.0***	22.9 ± 2.8
WC (cm)	95.8 ± 5.3***	84.0 ± 6.9	87.7 ± 6.6***	76.8 ± 7.1	89.2 ± 7.2***	78.4 ± 8.1
HDL-C (mmol/L)	1.08 ± 0.25***	1.25 ± 0.31	1.16 ± 0.24***	1.39 ± 0.32	1.24 ± 0.28***	1.44 ± 0.37
LDL-C (mmol/L)	2.71 ± 0.76	2.72 ± 0.74	2.66 ± 0.71	2.62 ± 0.72	2.92 ± 0.82	2.93 ± 0.79
TG (mmol/L)	2.13 ± 1.50***	1.41 ± 0.99	2.09 ± 1.41***	1.09 ± 0.64	2.01 ± 1.28***	1.27 ± 0.76
TC (mmol/L)	4.66 ± 1.01	4.62 ± 0.94	4.66 ± 0.94	4.60 ± 0.92	5.07 ± 1.08	5.10 ± 1.02
FPG (mmol/L)	6.77 ± 1.84***	6.16 ± 1.73	6.30 ± 1.99***	5.39 ± 1.02	6.58 ± 1.80***	5.63 ± 1.14
SBP (mmHg)	138.04 ± 16.01***	129.23 ± 16.45	131.17 ± 18.96***	117.53 ± 13.21	137.96 ± 16.42***	124.12 ± 16.13
DBP (mmHg)	83.61 ± 10.09***	78.80 ± 10.35	82.31 ± 10.82***	73.88 ± 9.54	79.46 ± 9.75***	74.39 ± 9.64
Regular exercise (%)	41.7	45.3	40.9	40.2	41.9	39.6
Current smoking (%)	23.3	22.3	7.2	7.5	7.1	6.6
Current alcohol intake (%)	15.3	13.8	3.0	4.7	4.5	3.7

Continuous data are expressed as means ± SD, category data as indicated. *MS* metabolic syndrome; *Ht* height; *Wt* weight; *BMI* body max index; *WC* waist circumference; *HDL-C* high density lipoprotein cholesterol; *LDL-C* low density lipoprotein cholesterol; *TG* triglyceride; *TC* total cholesterol; *FPG* fasting plasma glucose; *SBP* systolic blood pressure; *DBP* diastolic blood pressure.

***p < 0.001 compared with subjects without MS.

### QUS assessment

The results of covariates with adjusted mean QUS parameters in participants with and without MS are shown in Table 2. The associations between QUS parameters and MS were not significant either before or after adjusted by different models in males as well as in premenopausal women whereas in postmenopausal women with MS the BMD was found lower than those without before or after age adjustment. SOS in postmenopausal women with MS was significantly lower than in those without. However, after adjusted for age the difference regarding SOS disappeared. No differences regarding BUA, QUI or T-score were found before any adjustment between the groups in postmenopausal women, while the associations were significant after age adjustment.

**Table 2:** QUS parameters grouped by sex, menstrual states and MS states

	**With MS**	**Without MS**	**P value**			
			**Unadjusted**	**Model1**	**Model2**	**Model3**
Males						
BUA (dB/MHz)	75.92 ± 15.94	75.75 ± 16.73	0.788	0.831	0.957	0.962
SOS (m/s)	1539.56 ± 28.34	1540.89 ± 29.40	0.248	0.245	0.269	0.233
QUI/sti	91.37 ± 17.44	91.75 ± 18.47	0.593	0.579	0.559	0.492
BMD (g/cm^2^)	0.501 ± 0.111	0.503 ± 0.117	0.627	0.613	0.317	0.276
T-score	-0.953 ± 1.270	-0.926 ± 1.178	0.569	0.551	0.523	0.447
Premenopausal women						
BUA (dB/MHz)	77.52 ± 15.50	79.35 ± 15.26	0.110	0.088	0.355	0.322
SOS (m/s)	1547.23 ± 26.37	1549.81 ± 28.55	0.198	0.154	0.610	0.585
QUI/sti	95.15 ± 16.57	96.86 ± 17.48	0.184	0.143	0.476	0.446
BMD (g/cm^2^)	0.526 ± 0.104	0.537 ± 0.109	0.163	0.117	0.485	0.453
T-score	-0.475 ± 1.062	-0.353 ± 1.095	0.133	0.095	0.392	0.364
Postmenopausal women						
BUA (dB/MHz)	70.55 ± 16.88	71.38 ± 16.85	0.137	0.021^*^	0.851	0.825
SOS (m/s)	1531.68 ± 27.98	1533.68 ± 27.93	0.030^*^	0.081	0.295	0.299
QUI/sti	85.95 ± 17.74	87.00 ± 17.60	0.072	0.023^*^	0.474	0.489
BMD (g/cm^2^)	0.467 ± 0.113	0.474 ± 0.111	0.043^*^	0.041^*^	0.585	0.599
T-score	-1.030 ± 1.437	-0.987 ± 1.116	0.301	0.022^*^	0.370	0.385

Model 1 is adjusted by age, Model 2 is adjusted by age + BMI + WC, Model 3 is adjusted by age + BMI + WC + daily exercise + current smoking + current alcohol consumption. *BMI* body max index; *WC* waist circumference; *BUA* broadband ultrasound attenuation; *SOS* speed of sound; *QUI* quantitative ultrasound index; *BMD* bone mineral density.

^*^P < 0.05.

Stepwise multiple linear regression analysis with QUS parameters as the dependent variable and age, BMI, components of MS, alcohol intake, smoking, and regular exercise as independent variables was performed in each group. As shown in Table 3, in males, BMI was positively associated while WC was negatively associated with all QUS parameters. Positive relationship was also observed between FPG and SOS, QUI. In premenopausal women, only HDL-C had positive association with all QUS parameters, no significant association was found for any other components of MS. In postmenopausal women, we found age related negatively to all QUS parameters and BMI, HDL-C, FPG related positively to all QUS parameters. There were negative associations between WC and SOS as well as QUI. Positive relationship was also found between SBP and SOS, and the same between TG and QUI.

**Table 3:** Stepwise multiple linear regression analyses with QUS parameters as the dependent variable and age, BMI, components of MS (WC, HDL-C, TG, FPG, SBP, DBP), alcohol intake, smoking, and exercise as independent variables

	**Beta**		
	**BUA**	**SOS**	**QUI**
Males			
BMI	0.115^***^	0.150^***^	0.138^***^
WC	-0.093^**^	-0.192^***^	-0.153^***^
FPG	-	0.054^**^	0.048^*^
Premenopausal women			
HDL-C	0.123^***^	0.100^**^	0.111^***^
Postmenopausal women			
Age	-0.276^***^	-0.290^***^	-0.292^***^
BMI	0.085^***^	0.116^***^	0.109^***^
WC	-	-0.094^***^	-0.054^*^
HDL-C	0.065^***^	0.034^*^	0.058^**^
TG	-	-	0.036^*^
FPG	0.037^***^	0.053^**^	0.050^**^
SBP	-	0.047^**^	-

*BMI* body max index; *WC* waist circumference; *HDL-C* high density lipoprotein cholesterol; *TG* triglyceride; *FPG* fasting plasma glucose; *SBP* systolic blood pressure; *BUA* broadband ultrasound attenuation; *SOS* speed of sound; *QUI* quantitative ultrasound index.

^*^P < 0.05; ^**^P < 0.01; ^***^P < 0.001.

Besides, the number of MS components was not statistically associated with QUS parameters in both genders before or after age adjustment (data not shown).

### Fragile fractures

Of all 7489 participants, there were 9.59% (men) and 9.65% (women) subjects had fractures. Osteoporotic fractures were found in 3.84% men and in 5.95% women, respectively. Among the people who had MS, 6.8% women and 3.7% men have suffered from fragile fractures. Women with MS got higher incidence of fragile fractures than those without (6.8% VS. 5.3%, P = 0.034), while no impact of MS states on fragile fractures was found in male.

### 25(OH)D levels

In postmenopausal women, we randomly chose 1739 subjects who participated the project from September to November to have their serum 25(OH)D measured. We found that postmenopausal women with MS had lower 25(OH)D levels than those without (42.31 ± 14.07 nmol/Lvs.44.95 ± 14.63 nmol/L, P < 0.001), and the difference remained even after age and BMI adjustment. In women with vitamin D deficiency [25(OH)D < 50 nmol/L], the prevalence of MS was 54%, which was significantly higher than those without vitamin D deficiency (47.8%).

## Discussion

We have studied the relationship between MS and parameters of QUS in a relatively large middle-aged and elderly Chinese population. For the first time, the impact of the MS on osteoporotic fractures in this population was investigated. On one hand, QUS was chosen as an evaluation of bone characteristics because its convenience compared with DXA, on the other hand, MS represents a major health problem nowadays. More information provided by our study will help to understand the pathogenesis of metabolic bone disease further.

In our study, 39.7% participants were diagnosed with MS according to the IDF criteria, which was a little higher than the results from previous studies in China, mainly because our study is a community based study instead of epidemiological investigation and we did not standardize age and gender, and the mean age of subjects in our study is relative older. Besides, different diagnostic criteria may also lead to the difference.

To our knowledge, this is the first study showing an inverse association between MS and bone states assessed by QUS. For previous studies, the relationship between MS and QUS is not confirmed. Two studies in Spain found positive associations between MS and QUS [13,15], while a study in Taiwan [14] found no association between bone mineral loss and the MS in both genders. This is not surprising, for there is also a debate about the correlation between MS and BMD measured by DXA. This inconsistency may because of different population studied and different diagnostic standard used for MS. As far as we know, few studies used the same criteria as ours for investigating the impact of MS on QUS. Two reports which also choose IDF criteria for MS found different association between MS and BMD assessed by DXA. The study in Korean showed a negative association [17], whereas a Bulgaria study [18] confirmed the trend for higher BMD in men with MS.

Interestingly, the relationship between MS and QUS in this study differs between genders and between menopause statues. We did not found any relationship between MS and QUS in men and premenopausal women, which coincides with the Camargo cohort study [13] and the Taiwan study [14] about QUS, and also in agreement with previous studies with DXA [17]. A study in an older population even found that QUI was significantly lower in men with MS but significantly higher in women with MS when compared to their controls [16]. All these results suggest that bone metabolism changes in MS may be affected by a lot of factors coming from gender and menopause states which means different physiological situations.

We also investigated how different components of MS affect QUS in this study. BMI and WC were related to QUS parameters in men and postmenopausal women, people had higher QUS values with higher BMI and lower WC. The Camargo cohort study about QUS [13] and the studies by Cvijetic [16] and Kim [17] about ultrasound bone measurement and DXA, also got the similar conclusion, respectively. Together with their results [19,20], we support the idea that the relationship between obesity and osteoporosis depends on how obesity is defined. If obesity is based on BMI, it might protect against osteoporosis. However, obesity appears to be a risk factor for bone loss, if it is defined as central obesity. It is widely known that visceral fat is responsible for the storage and mobilization of lipids as well as releasing cytokines such as interleukin-6 (IL-6) and tumor necrosis factor-alpha (TNF-α), which play a role in bone remodeling via their effects on bone formation and resorption [19]. Thus, it is possible that central obesity in MS patients be related to bone loss, despite of the protective effects of weight. The diagnostic criteria for MS from IDF 2005 define the central obesity as the essential criterion, while some other diagnostic criteria do not necessarily include central obesity. So the weight of central obesity on bone metabolism may be bigger in our study as we use the IDF 2005 MS diagnostic system. This difference may also explain the disagreement between our study and others partially.

It is well known that vitamin D deficiency is a major risk factor of osteoporosis and it has been implicated in association with the development of diabetes, hypertension and obesity recently [21,22]. A five-year follow-up study in Australia found that lower 25(OH)D level was associated with increased MS risk [23], Maki et al. [24] found that greater dietary vitamin D intake was associated with reduced prevalence of MS, which was validated by us. Low 25(OH)D levels in MS population may partially explain why they have lower QUS and higher incidence of fragile fractures. The causes of why vitamin D deficient are associated with MS still need further investigation.

Concerning glucose intolerance, our study shows that impaired glucose tolerance seems to increase the QUS parameters in men and postmenopausal women. Sauque-Reyna et al. [25] also found T2DM is an independent protective factor for osteoporosis, using DXA. Besides, Bulló et al. [15] showed that individuals with T2DM had a significantly higher BUA and QUI than their non-diabetic counterparts. Most researchers agree that T2DM has increased bone mass but decreased bone quality contributing to increased risk of fractures [26].

Our results support what were found by Jeon et al. [27] about HDL-C level and BMD, suggesting a protective effect of this anti-atherosclerotic lipid on bone formation in women. While Buizert et al. [28] observed inverse association between QUS and HDL-C. We only found TG was positively associated with QUI in postmenopausal women, consistent with study in Turkey [29]. Some other studies suggest a certain relation between LDL-C and QUS which was not discovered by us.

It is interesting that in our study no obvious relation was observed between hypertension and QUS parameters, except slightly positive association between SBP and SOS in postmenopausal women. Till now, studies [13,14] about QUS and hypertension haven’t reached a confirmed conclusion.

According to our study, in subjects who had central obesity, most of them (44.03%) met two rest IDF MS diagnostic criteria, followed by 38.58% of those met three rest criteria, least subjects (17.39%) had all of the disorders. It was disappointing that the number of MS components had no impact on QUS parameters, i.e. more abnormalities do not increase the chance of QUS changes. Different from our results, the number of MS components was positively associated with BMD measured by DXA in both genders in previous studies [30,31].

Fragile fractures are the severe consequence of osteoporosis, leading to the increase in disability and mortality, as well as enormous family, social and economic burden. Old age, smoking, alcoholic drinking, hypertension, diabetes mellitus, hyperlipidemia, elevated C-reactive protein are all considered associated with osteoporotic fractures [32,33]. MS contents several factors that may impact on osteoporotic fractures. Our results showed that women with MS were more prone to develop osteoporotic fractures than those without MS. This phenomena was consist with the difference of QUS parameters caused by MS state found in this study, suggesting MS an adverse factor for bone health. This results also verified indirectly the potency of QUS on prediction of fractures which has been assessed by Ning and et al. [34].

The strengths of our study lie in its large-scale and population-based design, including both Chinese males and females older than 40 years. Although there are a few similar studies, they were based either on other race or on a much smaller population [13,14]. Besides of this, our study not only analysed the association between MS and osteoporotic fractures, but also provided useful information on the relations between MS and QUS in premenopausal women when others mainly focused on mainly postmenopausal women or men.

However, several limitations of our study have to be addressed. First of all, due to the cross-sectional nature of the present study, we can neither draw any causal inference, nor extrapolate our results to other population. Further longitudinal studies are needed to clarify the relationships between MS and QUS. Secondly, the information on fractures were only collected by questionnaires instead of source documents and radiological examinations, and the QUS measurements were taken several months or even years after the fractures which might differ from the QUS conditions when the fracture occurred. However, according to the study by Njeh et al. [35], time elapsed since fracture is not clinically related to QUS evaluation. Thirdly, we did not measure the level of estrogen, follicle-stimulating hormone, total calcium, parathyroid hormone and bone turnover markers, which might affect QUS evaluation. However, we have excluded subjects who take calcium, vitamin D, hormone replacement therapy, etc.

## Conclusion

Our large scale population-based research is the first to report the association both between MS and QUS and between MS and osteoporotic fractures in middle-aged and older Chinese population, especially including pre- and postmenopausal women, and it’s also the first one to show that MS has detrimental effect on bone mass by QUS and osteoporotic fractures in postmenopausal women, and the effect might be driven by central obesity as well as vitamin D deficiency, highlighting the importance of screening the BMD in postmenopausal women with MS and supplying vitamin D to them. More prospective studies are needed to clarify the underlying causes of these associations. The effects and the magnitude of different components of MS on bone metabolism also need further precise investigation.

## Abbreviations

QUS: Quantitative ultrasound; MS: Metabolic syndrome; IDF: International Diabetes Federation; 25(OH)D: 25-hydroxyvitamin D; QUI: Quantitative ultrasound index; BUA: Broadband ultrasound attenuation; BMI: Body mass index; WC: Waist circumference; HDL-C: High density lipoprotein cholesterol; FPG: Fasting plasma glucose; TG: Triglycerides; T2DM: Type 2 diabetes mellitus; BMD: bone mineral density; DXA: Dual energy X-ray absorptiometry; LDL-C: Low density lipoprotein cholesterol; TC: Total cholesterol; SOS: Speed of sound; Ht: Height; Wt: Weight; SBP: Systolic blood pressure; DBP: Diastolic blood pressure.

## Competing interests

All authors of this study reported no conflicts of interests.

## Authors’ contributions

MS, MC, QF, ZZ, CM, JM and YS carried out the participated in the whole project. MC and MS drafted the manuscript and performed the statistical analysis. YD, WT, Xiaoping Huang participated in the design of the study. WH and TY conceived of the study, and participated in its design and coordination and helped to draft the manuscript. All authors read and approved the final manuscript.

## Pre-publication history

The pre-publication history for this paper can be accessed here:

http://www.biomedcentral.com/1472-6823/14/14/prepub
